# The need for post-operative vasopressor infusions after major gynae-oncologic surgery within an ERAS (Enhanced Recovery After Surgery) pathway

**DOI:** 10.1186/s13741-020-00158-0

**Published:** 2020-09-07

**Authors:** Michèle Bossy, Molly Nyman, Thumuluru Kavitha Madhuri, Anil Tailor, Jayanta Chatterjee, Simon Butler-Manuel, Patricia Ellis, Aarne Feldheiser, Ben Creagh-Brown

**Affiliations:** 1 Department of Anaesthesia and Intensive Care Medicine, Royal Surrey County Hospital NHS Foundation Trust, Egerton Road, Guildford, Surrey, GU2 7XX UK; 2 Faculty of Medicine, University of Southampton, Southampton, UK; 3 Department of Gynae-oncology Surgery, Royal Surrey County Hospital NHS Foundation Trust, Egerton Road, Guildford, Surrey, GU2 7XX UK; 4 Department of Clinical and Experimental Medicine, Faculty of Health and Medical Sciences, University of Surrey, Guildford, Surrey, UK; 5 Department of Anesthesiology, Intensive Care Medicine and Pain Therapy, Evang. Kliniken Essen-Mitte, Huyssens-Stiftung/Knappschaft, Essen, Germany; 6 Department of Anesthesiology and Intensive Care Medicine, Campus Virchow-Klinikum and Charité Campus Mitte, Charité – Universitätsmedizin Berlin, Berlin, Germany

**Keywords:** Vasopressors, Vasoconstrictors, Vasoplegia, Shock, Cancer surgery, Gynaecological oncology, Peri-operative medicine, Anaesthesia

## Abstract

**Background:**

Hypotension following major abdominal surgery is common, and once hypovolaemia has been optimally treated, is often due to vasodilation which can be treated with vasopressor infusions. There is unpredictability in the dose and duration of post-operative vasopressor infusions, and factors associated with this have not been determined.

**Methods:**

We present a case series of consecutive patients who received major gynae-oncology surgery delivered within an Enhanced Recovery After Surgery (ERAS) pathway at a single institution. Patients were electively admitted from theatre directly to the intensive care unit (ICU). Data was collected prospectively into electronic databases (Philips ICCA, Wardwatcher) and then retrospectively collated and appropriate statistical analyses were performed. In the absence of a consensus definition of vasoplegia, we, necessarily arbitrarily, chose a noradrenaline dose of > 0.1 mcg/kg/min at 08:00 on the first post-operative day. The rationale is that this would be more than would typically be expected to counteract the vasodilatory effects of epidural analgesia, which is commonly used at our institution.

**Results:**

Data was collected from 324 patients, all treated between February 2014 and July 2016. The average age was 67 years and 39% received neoadjuvant chemotherapy. The commonest tumour type was ovarian (58%). The median estimated blood loss was 800 ml and epidural analgesia was used in 71%. Fifty per cent received post-operative vasopressor infusions: factors associated with this included epidural use and estimated blood loss. Nineteen per cent met our criteria for vasoplegia: factors associated with this included CRP on post-operative day 1 and P-POSSUM morbidity score. Hospital and ICU length of stay was prolonged in those who had vasoplegia.

**Conclusions:**

Patients commonly receive vasopressors following major gynae-oncologic surgery, and this can be at relatively high doses. Clinical factors only accounted for a minority of the variability in vasopressor usage—suggesting considerable biological variability. Optimal care of patients having major abdomino-pelvic surgery may include advanced haemodynamic monitoring and ready availability of infused vasopressors, in a suitable environment.

## Background

Hypotension following surgery is common, and as it is associated with the potential for harm, it requires prompt evaluation and treatment (McEvoy et al. 2019). Clinical management typically focuses on the optimisation of intravascular volume to exclude hypovolaemia (includes goal-directed fluid therapy, GDFT (Sun et al. 2017)), which may be due to fluid redistribution, or fluid losses—including bleeding. Acute myocardial dysfunction is rare, but once excluded, systemic vasodilation remains the probable cause.

Systemic vasodilation following surgery is commonly due to residual effects of anaesthetic agents (in the early stages), neuraxial blockade (epidural analgesia) or the systemic inflammatory response caused by surgery (Lambden et al. 2018; Choileain and Redmond 2006; Kohl and Deutschman 2006). Infused vasopressor (or vasoconstrictor) drugs counteract the vasodilation so that, for the same cardiac output, there is improved mean arterial pressure (MAP). Avoiding hazardously low systemic blood pressure preserves organ perfusion.

Vasoplegia is a term that refers to profound systemic vasodilation and is rarely described in the context of non-cardiac surgery. There is no consensus definition, and it is unclear whether it is a pathophysiologically distinct entity representing uncontrolled failure of vascular homeostasis or if it represents the end of a spectrum of vasodilation (Lambden et al. 2018).

Major abdomino-pelvic surgery for gynaecological cancer resection (gynae-oncology surgery, GOS) may include hysterectomy, oophorectomy, omentectomy, colectomy, removal of the lymph nodes and peritoneal stripping. Surgery is carried out through a large laparotomy incision, may be prolonged and is often associated with significant blood loss. The most common reason for this surgery is ovarian cancer, and completeness of tumour removal is associated with improved survival (Jayson et al. 2014).

Our institution is a tertiary referral centre for such surgery and all patients follow an Enhanced Recovery After Surgery (ERAS (Nelson et al. 2016a; Nelson et al. 2016b)) pathway which includes routinely receiving intra-operative GDFT (with repeated 250 ml boluses of balanced crystalloid until the nominal stroke volume, measured with a LiDCO, is less than 10%) and are transferred directly from the operating room to the intensive care unit (ICU) where GDFT continues and, if required, infused vasopressors are used to maintain an individualised MAP (typically between 65 and 75 mmHg). Epidural analgesia is offered to the large majority of patients.

This study was prompted by our observation of unpredictable variability in the required dose and duration for PVI. Currently, clinical variables like epidural use, blood loss and volume replacement are expected to be associated with PVI. Additional factors like hypoalbuminaemia, anaemia and recent chemotherapy are also hypothesised to confer an association. Yet, none of these factors has been studied. Even after considering all relevant clinical variables, there may be residual variability, perhaps relating to differences in biological response to surgical stimulus. We aimed to assess the association between all clinical variables and PVI as such might guide risk stratification and risk-adjusted destination for early post-operative care.

## Methods

The aim of this study was to describe the incidence of receipt of post-operative vasopressor infusions (PVI) in a cohort of patients who had undergone gynae-oncology surgery (GOS). Secondary aims included determination of factors associated with PVI, an estimate of the extent to which common clinical factors were able to determine PVI, and a description of the clinical outcomes.

The design was a retrospective review of prospectively acquired data, and the NHS Health Research Authority decision tool indicated that this did not require approval by a Research Ethics Committee. The setting of this study was the National Health Service (NHS) Royal Surrey County Hospital (RSCH), a tertiary surgical oncology centre in England. Data was collated and cross-referenced from several electronic records: The ICU electronic patient record (the IntelliSpace Critical Care and Anaesthesia, Phillips), ICU electronic patient database (WardWatcher, Critical Care Audit Ltd.) and a local bespoke system (the RSCH Gynae-Oncology database).

Patients included within the dataset were all consecutive patients undergoing elective GOS at the Royal Surrey County Hospital in Guildford between 4 February 2014 and 14 July 2016 (29.5 months). No patients were excluded. Patients that subsequently returned to theatre during the same hospital admission or underwent emergency surgery were not included again as part of this study.

In the absence of a consensus definition of vasoplegia and no published studies outside of cardiac surgery, we selected a noradrenaline dose of ≥ 0.1 mcg/kg/min at 08:00 on the first post-operative day as being indicative of vasoplegia. Our rationale is that this is more than would be typically expected to counteract the vasodilatory effects of epidural analgesia, which is commonly used at our institution (0.1% levobupivicaine with 2 mcg/ml fentanyl, typically at ~ 10 ml/h).

Data was collected on all available information considered relevant to the study aims, and this included patient age, weight, peak serum lactate level, date of surgery, histology of tumour and grade, surgical procedure, estimated blood loss (EBL), total fluid volume given, presence or absence of epidural and the rate of infusion, any infusion of phenylephrine or noradrenaline and the maximum dose, pre-operative haemoglobin and serum albumin levels, post-operative day one haemoglobin, albumin and CRP levels, length of ICU stay, length of hospital stay, level of care required, P-POSSUM morbidity and mortality scores, whether the patient received pre-op chemotherapy, APACHE II score and patient mortality.

Because of the sample sizes and deviations from normal distribution of the observations, data were expressed as median (25%, 75% quartiles) or frequencies (%), respectively. Differences with respect to continuous data were tested using the exact Mann-Whitney *U* test for independent groups, while frequencies were tested by the Fisher exact test. Associations of risk factors and the binary outcome of any vasopressor administration and presence of vasoplegia were evaluated using a multiple logistic regression model. To estimate effects, the odds ratio (OR) was indicated with a 2-sided confidence interval of 95% (95% CI), where appropriate.

A 2-tailed *p* value < 0.05 was considered statistically significant. The data was analysed using The R project for statistical computing, Version 3.0.1, Prism (GraphPad, Version 7), and IBM SPSS Statistics, Version 21, Copyright 1989, 2010 SPSS Inc.

## Results

During the period under consideration, February 2014 and July 2016, data from a total of 324 consecutive patients were collected. The average age was 66.5 (IQR 56–74) years and neoadjuvant chemotherapy was used in 39% of patients. Predicted morbidity and mortality were high. Table 1 summarises the patient’s pre-operative data.

**Table 1:** Patient pre-operative characteristics. Data are shown as median (25%; 75%) quartiles or as *n* (%) patients

Pre-operative; number of patients = 324	
Age (years)	66.5 (56–74)
Weight (kg)	69 (60–82)
Haemoglobin (g/l)	122 (109–134)
Albumin (g/l)	42 (40–44)
Tumour type, *n* (%)	
Ovarian	244 (75.30%)
Benign	31
Borderline	6
Malignant	207
Endometrial/uterine	62 (10%)
Cervix	2 (0.61%)
Vulva	2 (0.61%)
Vagina	1 (0.31%)
Bowel	6 (1.85%)
Other (GI/breast)	4 (1.23%)
Unknown primary	3 (0.93%)
P-POSSUM predicted risk of mortality (%)	5.8 (1.7–16.9)
P-POSSUM predicted risk of morbidity (%)	74.1 (38.5–92.5)
Neoadjuvant chemotherapy, *n* (%)	126 (39%)

The incidence of receiving any infusion of vasopressor was 50%, and the incidence of receiving infused noradrenaline at an infusion rate of at least 0.1 mcg/kg/min on the morning of post-operative day 1 was 19%. The median APACHE II score was 12 (9–15). However, the median duration of hospital stay was only 5 (4–7) days. Details are in Table 2.

**Table 2:** Details of intra- and post-operative events and outcomes. Data are shown as median (25%; 75%) quartiles or as *n* (%) patients

Intra-operative; number of patients = 324	
Estimated Blood Loss (ml)	800 (321–1400)
Total volume of IV fluid (ml)	2500 (1500–3500)
Transfused (n, %)	34 (11%)
Post-operative; number of patients = 324	
Cumulative fluid balance (l)	2.8 (1.6–4.7)
Epidural use	229 (70.67%)
Received any post-operative vasopressor infusion PVI, *n* (%)	161 (50%)
Met criteria for vasoplegia, *n* (%)	61 (19%)
Peak lactate in first 24 h, mmol/l	2.0 (1.4–2.9)
APACHE II score (points)	12 (9–15)
ICU LOS (days)	2 (1–3)
Hospital LOS (days)	5 (4–7)
Mortality at 28 days (%)	5 (1.54%)

Peri-operative patient characteristics split according to whether they fulfilled criteria for vasoplegia, or not, are presented in Table 3. There was no difference in pre-operative haemoglobin or albumin concentration between the two groups. Patients who developed vasoplegia weighed less than those who did not.

**Table 3:** Peri-operative patient characteristics split according to whether they fulfilled criteria for vasoplegia, or not

	No vasoplegia, median IQR (*n* = 259)	Vasoplegia, median IQR (*n* = 61)	*p* value
Pre-op			
Age (years)	66 (55.25–74)	70 (58–77)	0.209
**Weight (kg)**	**70 (60–83)**	**61 (54.5–70)**	**<0.001**
Pre-op Hb (g/l)	124 (111–134.75)	119 (104–131)	0.053
Pre-op albumin (g/l)	42 (39–44)	42 (39.25–44)	0.433
Pre-op chemotherapy no. (%)	78 (37%)	20 (39%)	0.872
**P-POSSUM mortality**	**4.85(1.7–13.2)**	**16.9 (7.0–23.6)**	**<0.001**
**P-POSSUM morbidity**	**70.9 (37.0–88.7)**	**92.4 (80.2–95.3)**	**<0.001**
Intra-op			
**IV fluid given (ml)**	**2000 (1275–3000)**	**3500 (2500–4650)**	**<0.001**
**Blood transfusion (ml)**	**0 (0–0)**	**0 (0–268.5)**	**0.019**
**Surgical_EBL (ml)**	**600 (300; 1200)**	**1450 (925–2000)**	**<0.001**
Post-op			
**Cumulative fluid balance (ml)**	**2760 (1572–4186)**	**4675 (1854–6549)**	**0.001**
Hb on POD1 (g/l)	108 (99–118)	111 (100.5–118)	0.396
**Hb drop (g/l)**	**17 (6–24.8)**	**9 (− 7–21)**	**0.01**
**Albumin POD1 (g/l)**	**29.5 (26–32)**	**27 (23–30)**	**<0.001**
**Albumin drop (g/l)**	**13 (9–15)**	**13 (11–17.8)**	**0.046**
**CRP POD1 (mg/l)**	**61 (40–95)**	**93 (48.5–131)**	**0.002**
Epidural use, no. (%)	179 (69%)	50 (82%)	0.058
**ICU LOS (days)**	**1.9 (1.05–2.88)**	**2.94 (2.22–3.88)**	**<0.001**
**Hospital LOS (days)**	**5.08 (4.01–6.49)**	**6.8 (5.78–8.17)**	**<0.001**
Mortality at 1 year, no. (%)	10 (5%)	4 (8%)	0.485

Patients with vasoplegia had higher predicted risk of morbidity and mortality suggesting that they were predisposed to complications which may have manifest as, or contributed to, receipt of vasopressors. They had higher intra-operative blood loss and received more blood transfusion and a greater volume of IV fluid. This was associated with prolonged ICU and hospital length of stay.

Multiple logistic regression was performed to determine factors associated with any PVI and factors that were found to be independently associated included cumulative fluid balance, epidural use and estimated blood loss (Table 4).

**Table 4:** Factors associated with any post-operative vasopressor infusion by presenting odds ratios (ORs, 2.5; 97.5%) obtained from multiple logistic regression analysis. ORs are adjusted for all other covariates. *EBL* estimated blood loss

	Odds ratio	2.5%	97.5%	*p* value
Age (years)	1.023	0.9989	1.0485	0.0648
Weight (kg)	0.9926	0.9778	1.007	0.3199
Peak lactate (mg/ml)	1.1224	0.8803	1.4413	0.3561
**Cumulative fluid balance (ml)**	**1.0002**	**1.0000**	**1.0003**	**0.0113**
**Epidural use, yes vs. no**	**3.9377**	**2.0585**	**7.786**	**0.0004**
CRP on POD1 (mg/ml)	1.0049	0.9992	1.0109	0.0954
Hb drop (g/l)	0.9862	0.9679	1.0041	0.135
Albumin drop (g/l)	0.969	0.9106	1.0215	0.2771
**EBL (ml)**	**1.0005**	**1.0002**	**1.001**	**0.0084**
P-POSSUM morbidity (%)	1.0043	0.9907	1.0181	0.5354
Pre-op chemotherapy	1.0249	0.5755	1.8337	0.9336

Multiple logistic regression was performed to determine factors associated with vasoplegia and factors that were found to be independently associated included weight, CRP on post-operative day 1 and P-POSSUM morbidity score (Table 5). Contrary to expectations, receipt of neoadjuvant chemotherapy was not associated with vasoplegia.

**Table 5:** Factors associated with vasoplegia by presenting odds ratios (ORs, 2.5; 97.5%) obtained from multiple logistic regression analysis. ORs are adjusted for all other covariates

	Odds ratio	2.5%	97.5%	*p* value
Age (years)	1.0039	0.9767	1.0329	0.784
Weight (kg)	**0.9763**	**0.9546**	**0.9962**	**0.0266**
Peak lactate (mg/ml)	1.1151	0.881	1.4092	0.3568
Cumulative fluid balance (ml)	1.000	0.9999	1.0001	0.4414
Epidural use, yes vs. no	1.6782	0.7551	4.0181	0.2206
CRP on POD1 (mg/ml)	**1.0058**	**1.0001**	**1.0115**	**0.0469**
Hb drop (g/l)	0.9949	0.9761	1.014	0.5932
Albumin drop (g/l)	1.0063	0.9527	1.0727	0.8358
EBL (ml)	1.0001	0.9999	1.0003	0.5676
P-POSSUM morbidity (%)	**1.0201**	**1.0037**	**1.0386**	**0.0214**
Pre-op chemotherapy	1.6519	0.8522	3.2144	0.1367

To determine the limitations to the relationship between the collected data and the occurrence of vasoplegia, a multiple *R*-squared test was performed. This found that the model only accounted for 13.46%.

C-reactive protein (CRP) measured on post-operative day 1 was significantly different between the groups. To further explore this relationship, a receiver operating characteristic (ROC) curve analysis was performed using two different cut-offs (Table 6). An elevated CRP level on post-operative day 1 had a significant prognostic power for a prolonged length of stay, as defined as > 75th centile or greater than 6.8 days. This indicates that of those patients with a CRP of more than 100; 80% of them will have a prolonged LOS on ICU.

**Table 6:** ROC curve for prolonged hospital length of stay

CRP (mg/l)	Sensitivity	Specificity
≥ 80	0.6	0.7
≥ 100	0.4	0.8

**Fig. 1:**
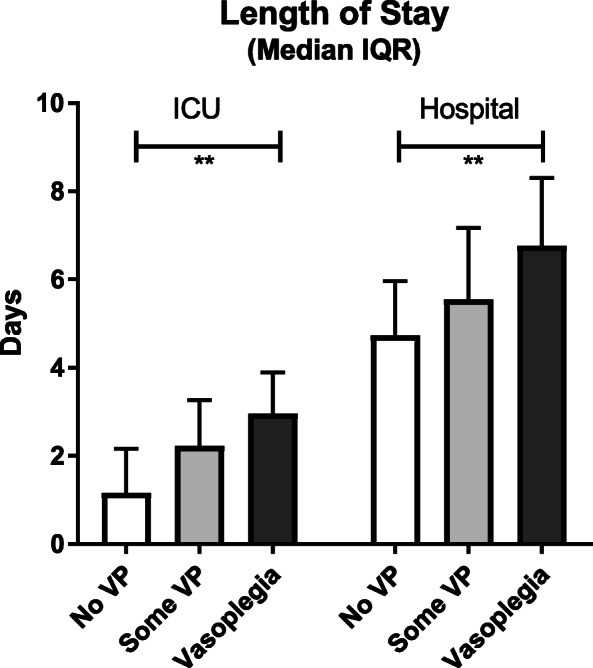
Length of stay in ICU and in hospital according to receipt of post-operative vasopressors. White boxes, patients who received no PVI; dark grey, met criteria for vasoplegia; light grey, received a PVI but did not meet criteria for vasoplegia. *p* < 0.001 Kruskal-Wallis test comparing ICU LOS between the three groups and equally *p* < 0.001 for hospital LOS between the three groups

The length of stay (LOS) in ICU and in hospital varies according to whether the patient had received no PVI, some PVI or if they fulfilled the criteria for vasoplegia; this is demonstrated in Fig. 1.

## Discussion

This study demonstrates that post-operative vasopressor usage is common (50%) in this cohort of patients, and although vasoplegia (as defined here) is relatively uncommon (19%), it is associated with increased length of stay in both the ICU and the hospital. These incidences were demonstrated despite being treated within an Enhanced Recovery After Surgery pathway and being optimised with advanced haemodynamic monitoring and goal-directed therapy.

We aimed to determine factors associated with PVI and found that epidural use, significant bleeding and cumulative fluid balance were associated. These associations are consistent with expectations, with cumulative fluid balance largely reflecting intravenous fluid administration. The clinical suspicion that pre-operative low haemoglobin, low albumin and receipt of neoadjuvant chemotherapy were associated with PVI was not supported by these data.

Receipt of post-operative vasopressor infusions following GOS has not been previously described in the literature. An analysis of a single-centre study in GOS patients described similar patient characteristics and clinical management (GDFT and use of epidural analgesia), and although there was a description of noradrenaline usage, it was limited to the intra-operative period (Hunsicker et al. 2015) and therefore no description of incidence of PVI.

### Strengths and limitations

The main strength of this study is reflected in its large, contemporaneous and consecutive cohort of relatively homogeneous patients receiving care in a single institution with standardised peri-operative care (ERAS pathways) and dedicated consultant-led teams providing the care. Despite the substantial uncertainty about optimal usage of intravenous fluid therapy (intra-op and post-op) (Sun et al. 2017; Restrictive versus liberal fluid therapy for major abdominal surgery | NEJM n.d.; Pearse et al. 2014), the management within this cohort was uniform. However, as a single-centre study, our findings should be interpreted with caution.

There is also no consensus definition for the appropriate use of post-operative vasopressors, or of vasoplegia. Although necessary, the arbitrary selection of a cut-off noradrenaline dose for this study may be disputable.

The study did not capture morbidity data, and any future prospective study should use standardised definitions (COMPAC-StEP (Myles et al. 2016)). The prolonged length of stay that was associated with receipt of vasopressors may be due to a range of factors, including de novo morbidity. For example, an episode of atrial fibrillation with associated hypotension might require treatment with a vasopressor and lead to a prolonged hospital stay. Without morbidity data, we cannot determine if it was the receipt of vasopressors per se that caused the prolongation in hospital stay or a complication that required the use of vasopressors. A high P-POSSUM morbidity score was associated with vasoplegia, but without data on complications, this is hard to interpret. The association may reflect that more extensive surgery (distant metastases and higher estimated blood loss are variables in the P-POSSUM score) causes more systemic inflammation that is manifested as vasodilation and reflected in prolonged receipt of vasopressors. A high POSSUM score could also suggest a predisposition to complications which could manifest as or contribute to the receipt of vasopressors.

A major determinant of vasopressor usage is the specified target—MAP targets are individualised to each patient, but there is subjectivity and therefore variability between clinicians. For example, if a MAP target is 75 mmHg, then the use of PVI is likely to be higher than if the target was 65 mmHg. This is an ongoing area of contention in critical care and peri-operative medicine (Dünser et al. 2016; Futier et al. 2017). A history of pre-existing cardiac disease may require higher MAP targets. Although none of the patients in this cohort was suspected of having peri-operative myocardial ischaemia, our study did not collect data on the prevalence of existing cardiac co-morbidities.

The relationship between weight and vasopressor infusion rate may be artefactual. The rationale for using a weight-based dosing regimen (rate of infusion of noradrenaline is typically given in micrograms per kilogramme of patient weight per minute, mcg/kg/min) assumes a linear relationship between patient weight and amount of norepinephrine required to achieve haemodynamic goals. The assumption is unlikely to be valid, specifically in the obese patient population because vasopressors are hydrophilic compounds that distribute poorly into fat (Wong et al. 2017). Patients who have increased adiposity will therefore have a lower volume of distribution relative to their weight and will appear to require a lower noradrenaline infusion rate than a patient with the same weight but less adiposity (Arabi et al. 2013). Any future prospective study should capture height to permit calculation of body mass index.

There may be significant variability in post-operative blood transfusion as the standard ICU practice is to avoid transfusion unless the haemoglobin concentration is less than 70 g/l, which contrasts with the practice of the gynae-oncologists who prefer significantly higher levels—particularly in patients who are due to receive adjuvant chemotherapy. Receipt of blood transfusion may have influenced the requirement for vasopressors.

### Clinical practice

The implications of this study for clinical practice is the recognition that patients who have major abdomino-pelvic surgery may require a period of haemodynamic support although this may be affected by epidural use, blood loss, fluid status or, perhaps, the existence of significant comorbidity like cardiac disease. Healthcare environments where such patients are routinely cared for on the ward after surgery, where infusions of vasopressors are not traditionally permitted, may be providing suboptimal care. Without the immediate availability of cardiac output monitoring and vasopressor infusions, patients may receive excessive volumes of IV fluids, which may be harmful (Voldby and Brandstrup 2016).

### Research recommendations

The paradigm of using repeated boluses of IV fluid until there is no further significant increase in stroke volume (fluid optimisation, GDFT), prior to starting vasopressors is being challenged. Controversially, earlier use of vasopressors is being explored in septic shock (Permpikul et al. 2019; Crystalloid liberal or vasopressors early resuscitation in sepsis n.d.), and if this proves to be superior to our current practice, this might also be true for peri-operative populations.

The recognition that there is considerable biological variability and that the detailed pathophysiology of post-operative vascular hyporeactivity has not been elucidated suggests there is scope to undertake translational studies in this area. Furthermore, such surgical cohorts with predictable vascular dysfunction may be considered as a resource to gain mechanistic insights into the underlying pathophysiology of vascular dysfunction more widely—perhaps as a model for septic shock.

This analysis needs to be replicated in other cohorts—other centres, other types of surgery and indeed other healthcare environments. The role of pre-operative factors (such as cardiac dysfunction) needs to be further determined and future work could focus on developing tools to risk stratify which patients are more likely to require vasopressor support following surgery. More generally, the epidemiology of post-operative vascular dysfunction has not been studied, but this will be partially addressed with an international prospective observational cohort study focused on receipt of post-operative vasopressor infusions, due to start recruiting in 2020, named ‘Squeeze’.

## Conclusions

In this cohort, patients commonly received vasopressors following major gynae-oncologic surgery, and in some patients, this was at higher doses or for days. Clinical factors that are associated include epidural use, bleeding, transfusion and use of IV fluids. However, these factors only account for a minority of the variability in vasopressor usage—suggesting the influence of biological variability and or unmeasured confounding variables such as pre-existing cardiac disease. Optimal care of patients having major abdomino-pelvic surgery may include advanced haemodynamic monitoring and the availability of infused vasopressors, in a suitable environment.

## Data Availability

Please contact author for data requests.

## Abbreviations

CRP: C-reactive protein EBL Estimated blood loss
GDFT: Goal-directed fluid therapy
GOS: Gynae-oncology surgery
ICU: Intensive care unit
LOS: Length of stay
MAP: Mean arterial pressue ERAS Enhanced Recovery After Surgery
NHS: National Health Service
PVI: Post-operative vasopressor infusion
RSCH: Royal Surrey County Hospital
